# Dysfunctional Endothelial Progenitor Cells in Metabolic Syndrome

**DOI:** 10.1155/2012/585018

**Published:** 2011-09-19

**Authors:** Sridevi Devaraj, Ishwarlal Jialal

**Affiliations:** ^1^Laboratory for Atherosclerosis and Metabolic Research, UC Davis Medical Center and the VA Medical Center, Sacramento, CA 95817, USA; ^2^Department of Pathology and Immunology, Baylor Medical Center, Houston, TX, USA

## Abstract

The metabolic syndrome (MetS) is highly prevalent and confers an increased risk of diabetes and cardiovascular disease. A key early event in atherosclerosis is endothelial dysfunction. Numerous groups have reported endothelial dysfunction in MetS. However, the measurement of endothelial function is far from optimum. There has been much interest recently in a subtype of progenitor cells, termed endothelial progenitor cells (EPCs), that can circulate, proliferate, and dfferentiate into mature endothelial cells. EPCs can be characterized by the assessment of surface markers, CD34 and vascular endothelial growth factor receptor-2, VEGFR-2 (KDR). The CD34^+^KDR^+^ phenotype has been demonstrated to be an independent predictor of cardiovascular outcomes. MetS patients without diabetes or cardiovascular diseases have decreased EPC number and functionality as evidenced by decreased numbers of colony forming units, decreased adhesion and migration, and decreased tubule formation. Strategies that have been shown to upregulate and enhance EPC number and functionality include statins, angiotensin converting enzyme inhibitors, angiotensin receptor blockers, and peroxisome-proliferator-activating-receptor gamma agonists. Mechanisms by which they affect EPC number and functionality need to be studied. Thus, EPC number and/or functionality could emerge as novel cellular biomarkers of endothelial dysfunction and cardiovascular disease risk in MetS.

## 1. Introduction: The Metabolic Syndrome

The metabolic syndrome (MetS) comprises a cluster of abnormalities, with insulin resistance (IR) and adiposity as central features [1]. Five diagnostic criteria have been identified by the National Cholesterol Education Program Adult Treatment Panel III (NCEP-ATP III), and the presence of any three features (central obesity, dyslipidemia [high triglycerides, low HDL], hypertension, and impaired fasting glucose) is considered sufficient to diagnose the syndrome. Approximately 35% of US adults have the MetS and this appears to be a very common syndrome globally. Also, the prevalence increases with age [2]. MetS confers a two- to fourfold increased risk for cardiovascular disease (CVD) and fivefold increased risk of diabetes [3].

## 2. Endothelial Dysfunction and MetS

A key early event in atherosclerosis is endothelial cell dysfunction, which is precipitated by several noxious insults including obesity, hypertension, and dyslipidemia hyperglycemia, all features of MetS. Numerous groups have reported endothelial dysfunction in patients with MetS. Esposito et al. showed that compared with 60 control subjects matched for age and sex, patients with the metabolic syndrome had decreased endothelial function [4]. In the Framingham Offspring participants, Hamburg et al. [5] showed, in age and gender adjusted models, that MetS was associated with decreased flow-mediated dilation (FMD). There was progressively lower vasodilator function with increasing number of MetS components. Lteif et al., using leg blood flow measurements, showed that patients with MetS had worse endothelial function [6]. Also, in the Prospective Study of the Vasculature in Uppsala Seniors (PIVUS) study, using different techniques to assess vasodilation in conduit and resistance arteries in MetS, the authors showed decreased flow-mediated vasodilation (FMD) [7]. In the Northern Manhatten study (NOMA), Suzuki et al. reported that MetS was associated with decreased flow-mediated dilatation (FMD) and increased CVD over 81 months [8]. Thus, it is clear that MetS patients have impaired endothelial function. This has major implications with regards to subsequent CVD. 

However, despite being used in several studies, the measurement of endothelial function by flow-mediated dilation is far from optimum and there is much variability in the studies reported above such as the NOMA and Framingham studies in which the mean FMD in controls were 6.3 and 3.3%, respectively.

## 3. Endothelial Progenitor Cells (EPC)

There has been much interest recently in a sub-type of progenitor cells, isolated from bone marrow, umbilical vessels, and peripheral blood of adults that have the capacity to circulate, proliferate, and differentiate into mature endothelial cells, termed endothelial progenitor cells (EPCs). EPCs circulate in the blood and appear to home preferentially to sites of vascular or tissue injury, contributing significantly to both reendothelialization and neoangiogenesis. It needs to be stated at the outset that there is much controversy with respect to the correct definition of EPCs [9–11]. Generally, it is accepted that EPCs are characterized by the assessment of surface markers such as CD34 and vascular endothelial growth factor receptor-2, VEGFR-2 (KDR) [11]. Importantly, CD34^+^KDR^+^ combination is the only putative EPC phenotype that has been demonstrated repeatedly and convincingly to be an independent predictor of cardiovascular outcomes [12, 13].

## 4. EPC and Cardiovascular Events

In a 10-month follow-up study, Schmidt-Lucke et al. [14] showed that the level of CD34^+^KDR^+^ cells independently predicted cardiovascular events and progression of atherosclerosis in a mixed population of healthy subjects and cardiovascular patients. In a larger study, Werner et al. [15] have reported that CD34^+^KDR^+^ cell count predicted cardiovascular events and cardiovascular death during a 12-month followup in 519 patients with coronary artery disease (CAD). Also, in a subset, colony forming units (CFUs) predicted cardiovascular events. Furthermore, Hill et al. [16] reported a strong correlation between the number of circulating endothelial progenitor cells (measured as colony forming units (CFUs)) and the subjects' combined Framingham risk factor score. Also, the measurement of flow-mediated brachial-artery reactivity revealed a significant relation between endothelial function and the number of progenitor cells. Indeed, levels of circulating EPC were a better predictor of vascular reactivity than was the presence or absence of conventional risk factors. Fadini et al. showed that a low CD34 count, a measure of progenitor cells, in addition to metabolic syndrome was associated with increased cardiovascular events (CVEs) [17]. Fadini's group have also shown an association between EPC reduction and increased carotid intima media thickness (c-IMT), as a marker of early atherosclerotic remodeling in healthy subjects [18].

In addition to flow cytometric quantitation of CD34/KDR predicting CVE, also functional assays such as CFU and EPC migration have been shown to correlate significantly with CAD risk factors, severity, and events [12–16]. Thus, the measurement of EPCs may be a surrogate biologic marker for vascular function and cumulative cardiovascular risk, suggesting further that endothelial injury in the absence of sufficient circulating progenitor cells may unfavorably affect the progression of CVD.

Additionally, various risk factors for CVD have been shown to impair EPCs in terms of functional features: proliferation (important for amplifying the cellular pool), migration (critical for homing of circulating EPCs), and survival [19]. Furthermore, decrease in circulating EPCs contributes to impaired angiogenesis as well as progression of atherosclerosis and patients at risk for CAD have decreased number of circulating EPCs with impaired activity. Thus, it seems important that both the number and functional activity of EPCs should be investigated. The individual components of the MetS are associated with impairment of EPCs number and function [20].

## 5. EPC and MetS

With regards to the MetS, there is sparse data on EPC number and functionality [21]. There appears to be two studies that have directly looked at EPC number in MetS patients (without other confounding, comorbidities such as diabetes or cardiovascular disease) and matched controls. In the study by Westerweel et al., they show that circulating CD34^+^KDR^+^ EPC levels were reduced by nearly 40% in obese men with MetS compared to nonobese men [22]. Although this was a small study that included 19 patients with MetS, it is important to emphasize that in this study, they excluded patients with overt clinical CVD or diabetes. They did not study EPC functionality.

In the study by Jialal et al. [23], they reported on EPC number and functionality in a larger sample size of subjects with MetS (*n* = 46) of which 77% were female and matched controls (*n* = 31). In accord with the study in obese males, they showed a significant decrease in EPC number, also defined by CD34/KDR dual positivity. Furthermore, these investigators also looked at functionality of EPCs such as colony forming units, migration, and tubule formation [23]. In addition to the reduction in numbers, they showed that there were significant impaired clonogenic capacity and also an impaired capacity to incorporate into tubule structures. Whilst there was a decrease in migration of the EPCs in MetS this did not attain significance. However, it needs to be emphasized that none of the subjects were diabetic or had CVD in the above 2 studies and none were on medications that affect EPCs suggesting that the defect in EPCs manifest early in nascent MetS prior to the development of diabetes or CVD. Fadini et al. have reported in a study decreased circulating EPCs and progenitor cells in diabetic patients with peripheral vascular disease [24]. In this paper, they did a subgroup analysis of MetS patients versus non-MetS patients. However, not much detail is provided with respect to coexistent diseases and morbidity such as diabetes and peripheral vascular disease or concomitant medications in these two subgroups. Since, this was a study with the primary aim to look at EPC status in diabetic patients with peripheral vascular disease the data in patients in MetS is not as detailed as reported in the 2 studies that focused on MetS alone [22, 23]. In a subsequent report by Fadini et al., they showed that in patients with MetS, there was a decrease in progenitor cells (CD34^+^ cells) [17]. It appears that many of these patients also could have diabetes, and be on medications such as statins, angiotensin converting enzyme Inhibitors (ACE-I), angiotensin receptor blockers (ARBs), and antidiabetic therapy such as pioglitazone, which could have influenced the data [24]. The reported decrease in progenitor cells in these 2 studies was confirmed in the study by Jialal et al. [23]. Previously, Satoh et al. [25] have reported increased EPC number in CAD patients with MetS and without MetS. They did not compare patients with MetS with controls and their sample sizes were small (*n* = 15 for acute myocardial infarction and *n* = 16 for patients with stable angina angina pectoris and MetS, resp.). Interestingly, they also showed increased oxidative DNA damage, decreased telomerase activity, and decreased telomere length, a marker of increased senescence in EPCs of CAD patients with MetS than the CAD patients without MetS. This suggests that the increase in EPC with CVD was a dysfunctional population since EPCs are generally well endowed with antioxidant defenses. Other functional measures of EPC activity such as tubule formation or colony forming units or adhesion was not investigated in this study. Thus, this needs to be investigated further.

Recently, Vignera et al. [26] reported increased EPCs in patients with arterial erectile dysfunction and MetS compared to controls. It is possible that in ED, where there is profound vascular dysfunction, a particular subtype of EPC (CD45 negative, CD34 positive, and CD144 positive) are increased due to a compensatory increase in mobilizing factors and could depict repair mechanisms. It is however important to point out that these investigators did not use the classical CD34/KDR criteria. Furthermore, the increased EPC in their subjects correlated with endothelial microparticles (EMPs) and IMT suggesting that this is a dysfunctional population. However since they did not study EPC functionality unlike the study by Satoh et al. [25], one cannot critically appraise this report. Also, limited data is provided with respect to medications that can affect EPC numbers and comorbidities such as diabetes and CVD, which are common accompaniments of erectile dysfunction and could further influence their findings. Indeed, previous studies have also reported decreased EPCs in such patients and a significant correlation of the decreased EPCs to increased cardiovascular risk. 

There is very limited data that specifically looked at EPC status either number and/or functionality in patients with MetS without the complications of diabetes and CVD. In the two studies which specifically address this, both have shown, decreased number of EPC, they are at variance with respect to a decrease in progenitor cells since levels were not significantly lower in the study in obese males. However if the data from the studies by Fadini are considered one could conclude that progenitor cell exhaustion can be advanced as one mechanism resulting in decrease in EPC number [27]. In addition, in the Jialal et al. [23] study, they showed significant correlation of CRP levels in MetS with decreased EPC number and functionality, pointing to the role of inflammation in this process.

**Figure 1:**
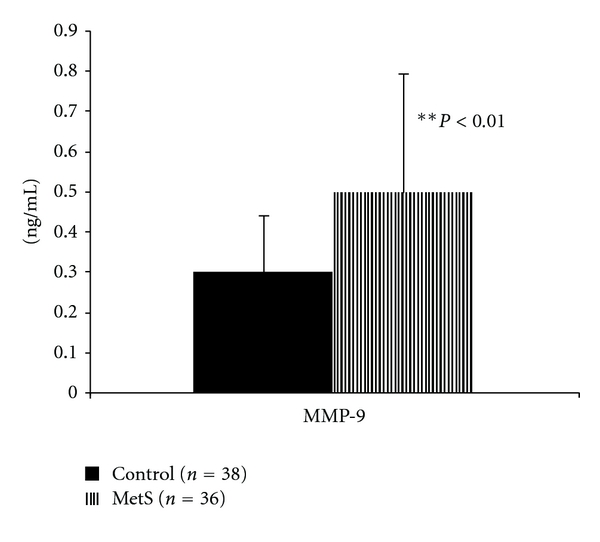
MMP-9 Levels in Patients with MetS compared to matched controls. Plasma MMP-9 levels were measured in patients with MetS compared to matched healthy controls (*n* = 38 and 36/group, resp.) using a sandwich ELISA (R&D Biosystems). Data are expressed as mean ± S.D in ng/mL. **P < 0.01 compared to controls.

There is limited data with regards to mobilizing factors in patients with MetS. Egan et al. [28] have reported the profound reduction in EPCs due to impaired mobilization from bone marrow because of the lower expression of CXC Chemokine Receptor 4 (CXCR4+)/CD34+ cells in diabetics versus controls. Importantly, CXCR4, CD117, and KDR are defined as the mobilizing receptors for progenitor cells (PCs) [29, 30]. Thus, it appears that the measurement of the respective circulating ligands; vascular endothelial growth factor (VEGF) for KDR, soluble c-kit ligand (KitL) for CD117, and Stromal derived factor 1 (SDF-1) for CXCR4 is also important. Some of the accepted mobilizing factors include VEGF, stromal derived factor-1 (SDF-1), and c-kit ligand [30]. In a small study that has examined mobilizing factors in MetS, the investigators showed no significant differences in VEGF levels, but showed that progenitor cell mobilizing stromal cell-mobilizing factor (SCF) and the soluble form of SCF receptor c-kit were both reduced in patients with MetS [22]. Since this is a limited study in a small number of patients, these findings need to be confirmed in a larger study. Jialal et al. [31] recently showed in subjects with MetS (*n* = 36) compared to age- and gender-matched controls (*n* = 38) that there was a significant reduction of 83% in granulocyte colony-stimulating factor levels in patients with MetS. Also, there were decreases in SCF and SCF soluble receptor levels. However, there was no significant difference in stromal cell-derived factor-1 levels, and paradoxically, vascular endothelial growth factor levels were increased, consistent with VEGF resistance, which has been reported previously with insulin resistant states such as diabetes and MetS [32]. Data on matrix metalloproteinase (MMP)-9 levels in patients in MetS is sparse. Previously, MMP-9 levels have been shown to be increased in subjects with MetS using immunoassay [33]. Since MMP-9 levels are critical for homing of EPC [34], we examined levels of MMP-9. In accord with the previous report we show an increase in MMP 9 levels by immunoassay (Figure 1). However the relevance of this finding is questionable since we did not assay enzyme activity by zymography which is the superior measure of MMP-9 activity.

Thus, whilst there is much controversy with regards to the nomenclature and definition of EPCs, it needs to be emphasized that EPC number, EPC migration, and colony forming units which appear to connote early EPCs have clearly been shown to correlate with risk factors, CVD severity and predict CVD events to date. However, whilst it is claimed that the late EPCs are more likely to become endothelial cells, it needs to be emphasized that to date no studies have reported that late EPCs predict CVE in patients [30]. 

Thus, the published studies have shown that EPC numbers and functionality is impaired in MetS. The potential mechanisms that have been advanced so far include decreased progenitor cells and dysregulation of EPC mobilizing factors. Longevity of EPC in MetS has not been reported and thus studies directed at telomere biology and apoptosis are urgently needed in patients with MetS without comorbidities.

In conclusion, EPC number and functionality could serve as an additional novel cellular biomarker of endothelial integrity and impaired neoangiogenesis in patients with MetS who clearly have manifest endothelial dysfunction. Prospective studies should demonstrate that they predict CVD. Strategies that have been shown to upregulate and enhance EPC number and functionality such as statins, ACE-I, ARBs, PPAR-gamma agonist, and INCRETIN-based therapies, need to be studied more carefully with respect to both number and functionality of EPCs since this could inform us of their direct beneficial effects on the vulnerable vasculature of Mets. Thus, EPC number and/or functionality could emerge as a novel cellular biomarker of CVD risk and could better inform clinicians about potential pharmacotherapy for patients with MetS.
